# Prenatal Diagnosis and Genetic Analysis of a Fetus with Joubert Syndrome

**DOI:** 10.1155/2018/7202168

**Published:** 2018-05-31

**Authors:** Jingjing Xiang, Lili Zhang, Wei Jiang, Qin Zhang, Ting Wang, Haibo Li, Hong Li

**Affiliations:** ^1^Center for Reproduction and Genetics, The Affiliated Suzhou Hospital of Nanjing Medical University, Suzhou, Jiangsu, China; ^2^Center for Reproduction and Genetics, Suzhou Municipal Hospital, Suzhou, Jiangsu, China; ^3^Center for Medical Ultrasound, The Affiliated Suzhou Hospital of Nanjing Medical University, Suzhou, Jiangsu, China; ^4^Center for Medical Ultrasound, Suzhou Municipal Hospital, Suzhou, Jiangsu, China

## Abstract

**Objective:**

To diagnose and explore the genetic cause of Joubert syndrome (JS) in a fetus.

**Methods:**

Prenatal ultrasound and magnetic resonance imaging (MRI) examinations were performed, and genetic analysis was conducted using targeted next-generation sequencing (NGS) and Sanger sequencing.

**Results:**

Prenatal ultrasound and MRI examinations showed cerebellar vermis hypoplasia and molar tooth sign (MTS); hence the fetus was diagnosed with JS. Further genetic analysis revealed a known missense variant (c.3599C>T, p.A1200V) and a novel missense variant (c.3857G>A, p.R1286H) in the* C5orf42* gene of the fetus.

**Conclusion:**

Our study provides insights into prenatal and early diagnosis of JS and expands the variation spectrum of* C5orf42* gene.

## 1. Introduction

Joubert syndrome (JS, MIM 213300) is a rare neurodevelopmental disorder first described by Joubert in 1969 [1]. The incidence rate of JS is estimated between 1/80,000 and 1/1,00,000 live births [2]. JS is clinically heterogeneous, and the key clinical features of JS consist of cerebellar and brain stem malformation called the molar tooth sign (MTS) [3], hypotonia, and developmental delay/intellectual disability. Associated clinical findings of JS include cystic kidney disease, retinal dystrophy, hepatic fibrosis, and polydactyly. Therefore, JS is categorized into six phenotypic subtypes: classic or pure JS; Joubert syndrome with retinal disease (JS-Ret); Joubert syndrome with renal disease (JS-Ren); Joubert syndrome with oculorenal disease (JS-OR); Joubert syndrome with hepatic disease (JS-H); and Joubert syndrome with oral-facial-digital features (JS-OFD) [4, 5].

JS is also genetically heterogeneous as 34 causative genes have been identified to date, of which 33 genes are autosomal recessive and one gene (*OFD1*) is X-linked [4]. Most of the genes encode proteins known or predicted to be involved in the function of the primary cilium or basal body. The primary cilium is microtubule-based and involved in a wide variety of cellular processes, and its dysfunction could cause various human diseases collectively categorized as “ciliopathies” [6]. Molecular diagnosis of JS is challenging due to its genetic heterogeneity; however, the advent of next-generation sequencing (NGS) in the last few years has revolutionized genetic studies, which could help to identify the genetic causes, provide more accurate information for understanding genotype-phenotype correlations, and aid in genetic counseling, diagnosis, prognosis, and treatment.

In this study, we described the prenatal diagnosis and clinical features of a fetus with JS by ultrasound and magnetic resonance imaging (MRI) examinations. Further genetic analysis using targeted next-generation sequencing (NGS) and Sanger sequencing revealed that two compound heterozygous variants in the* C5orf42* gene might be responsible for this disorder.

## 2. Patient and Methods

### 2.1. Patient

This study was approved by the Institutional Ethics Committee of the Affiliated Suzhou Hospital of Nanjing Medical University. Written informed consent was obtained from all the individuals who attended this study. Parental consent was obtained from the children who are under 18 years of age. All the individuals of the family were subjected to comprehensive physical examination and full medical history evaluation. A pedigree of their family was created after clinical examination and genetic testing of all available family members.

### 2.2. Targeted Next-Generation Sequencing and Data Analysis

Genomic DNA was extracted from the fetal skin after autopsy using the QIAamp DNA Mini Kit (Qiagen, Germany) according to the manufacturer's procedures. Targeted next-generation sequencing (NGS) was applied using the Agilent SureSelect XT Inherited Disease Panel containing 2,742 genes (Agilent Technologies, USA) and an Illumina HiSeq 2500 System (Illumina, USA). Data analysis was performed using NextGENe (SoftGenetics LLC, USA) and candidate variants were screened by Ingenuity Variant Analysis (Ingenuity Systems, USA) as described [7].

### 2.3. PCR Amplification and Sanger Sequencing

Peripheral blood was collected from all available family members after giving informed consent. Genomic DNA was extracted from the blood samples using the QIAamp DNA Blood Mini Kit (Qiagen, Germany) according to the manufacturer's procedures. To confirm the identified variants, exons 20 and 22 of* C5orf42* were amplified by polymerase chain reaction (PCR), respectively, using FastStart Taq DNA polymerase (Roche, Switzerland). The PCR amplification program included an initial denaturation at 94°C for 5 min, 16 cycles of denaturation at 94°C for 45 sec, and annealing at 68°C for 45 sec, with the annealing temperature decreasing by 0.5°C at each succeeding cycle, extension at 72°C for 45 sec, followed by 20 cycles of denaturation at 94°C for 45 sec, annealing at 56°C for 45 sec, extension at 72°C for 1 min, a final extension at 72°C for 7 min, and holding at 4°C. The amplified DNA fragments were purified and sequenced in both directions using ABI 3130 Genetic Analyzer. The resulting sequences were compared with the reference sequence of* C5orf42* (NM_023073.3) in the NCBI database.

### 2.4. *In Silico* Analysis of Variants

Multiple sequence alignment of the C5orf42 protein and its orthologs was performed using MUSCLE [8] (http://www.drive5.com/muscle/). The variants were analyzed according to the Standards and Guidelines for the Interpretation of Sequence Variants released by the American College of Medical Genetics and Genomics and the Association for Molecular Pathology [9]. The corresponding variants were searched in the dbSNP (http://www.ncbi.nlm.nih.gov/SNP/), Exome Aggregation Consortium (ExAC) (http://exac.broadinstitute.org/), the Genome Aggregation database (gnomAD) (http://gnomad.broadinstitute.org/), the 1000 Genomes Project database (http://www.1000genomes.org/), and the database of Chinese genomes in diseaseDX (http://diseasedx.virgilbio.com/). The pathogenicity of variants was predicted by PolyPhen-2 [10] (http://genetics.bwh.harvard.edu/pph2/) and PROVEAN [11] (http://provean.jcvi.org).

## 3. Results

### 3.1. Clinical Data

**Figure 1:**
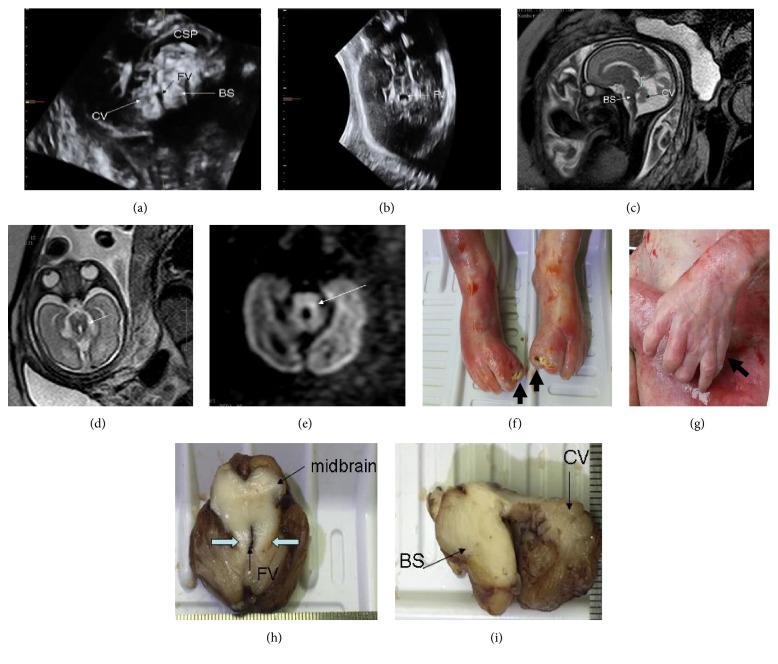
**Clinical features of the fetus.** (a) The sagittal ultrasound image shows hypoplasia of the cerebellar vermis and a triangular shaped fourth ventricle. (b) The axial ultrasound image shows a 'bat-wing'-shaped superior fourth ventricle. (c) The sagittal MRI image shows vermian agenesis and hypoplastic superior cerebellar peduncle. The axial T2-weighted (d) and diffusion-weighted (e) MRI images show prominent interpeduncular fossa and a deep cleft between thickened cerebellar peduncles comprising the molar tooth sign, as indicated by the arrows. After artificial abortion, polydactyly of both feet (f) and left hand (g) was noted, and the brain autopsy revealed the molar tooth sign (h, i). CV: cerebellar vermis, BS: brainstem, FV: fourth ventricle, CSP: cavum septi pellucidi.

The mother is a 31-year-old woman, and her husband is 36 years old. They are healthy and nonconsanguineous. The mother (“gravida 4, para 1”, G4P1) had four pregnancies and delivered a healthy girl ten years ago. She also experienced two induced abortions. Routine mid-trimester fetal ultrasound scan at 23^+4^ weeks of gestation suggested agenesis of cerebellar vermis, which was confirmed by a follow-up ultrasound scan at 29^+3^ weeks of gestation (Figures 1(a) and 1(b)). And fetal brain MRI performed at 29^+3^ weeks showed deep interpeduncular fossa and thick, elongated cerebellar peduncles, consistent with the MTS, as well as hypoplastic cerebellar vermis (Figures 1(c)–1(e)). Based on the results of ultrasound and MRI, the fetus was diagnosed with JS. The pregnancy was electively terminated at 29^+4^ weeks' gestation and autopsy was performed. The fetus displayed polydactyly of left hand and both feet, and the brain autopsy revealed the molar tooth sign, which confirmed the diagnosis of Joubert syndrome with oral-facial-digital features (JS-OFD) (Figures 1(f)–1(i)).

### 3.2. Genetic Analysis

**Figure 2:**
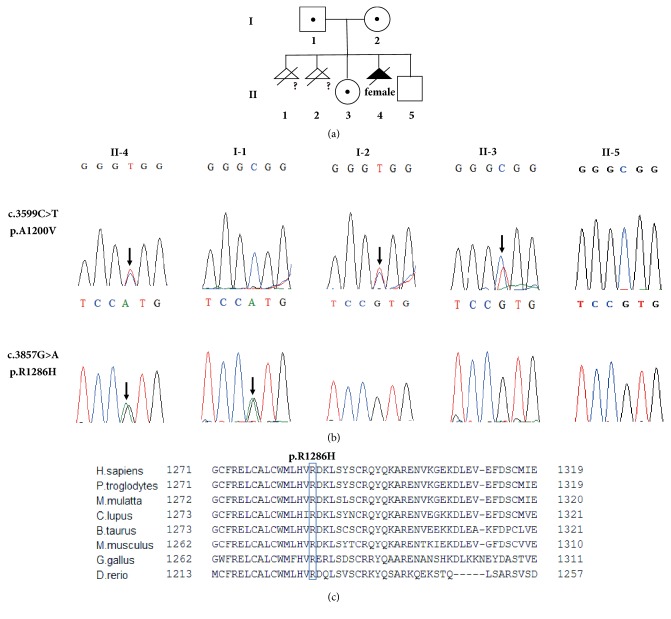
**Genetic analysis of the family.** (a) The pedigree of the family. Individuals marked with a question mark (?) were not genotyped for the* C5orf42* variants. (b) Sequencing chromatographs of* C5orf42 *gene revealed variants in the proband, the parents, and the sister. Variants were indicated by black arrows. (c) Sequence alignment of C5orf42 protein and its orthologs in different species. The amino acid in position 1286 is highlighted by a blue box.

JS causative genes were captured for targeted next-generation sequencing using the Agilent SureSelect XT Inherited Disease Panel, and the average read depth is over 100X. The candidate variants were screened using Ingenuity Variant Analysis. A missense variant (c.2524G>A, p.G842R) has been found in the* OFD1* gene, which is an X-linked causative gene of JS. However, this variant is inherited from the normal father and predicted to be benign by PolyPhen-2 (with a score of 0.116) and neutral by PROVEAN (with a score of -0.845), therefore it is excluded. Ultimately, two compound heterozygous variants in the* C5orf42* gene were identified. One variant was a heterozygous missense variant (c.3599C>T, p.A1200V) in exon 20, and the other was a novel heterozygous missense variant (c.3857G>A, p.R1286H) in exon 22. Direct sanger sequencing results confirmed the compound variants in the fetus and revealed that the father was heterozygous for the c.3857G>A variant, and the mother and sister were both heterozygous for the c.3599C>T variant (Figure 2(b)). A pedigree of this family was shown in Figure 2(a). The c.3599C>T variant has been identified in multiple patients with JS previously [12–14] and classified as pathogenic in the ClinVar database (https://www.ncbi.nlm.nih.gov/clinvar/variation/217591/). The c.3857G>A variant was recorded in the dbSNP (rs139464953) and showed low allele frequencies in the ExAC (T=0.0000169, 2/118316), gnomAD (T=0.00001806, 5/276920), 1000 Genomes Project database (T=0.000199, 1/5008, Release phase 3), and the database of Chinese genomes in diseaseDX (T=0.000155, 1/6468) but only in heterozygous state. Sequence alignment of the C5orf42 protein sequence in different species revealed that the arginine residue (R1286 in the human protein) is highly conserved among species (Figure 2(c)). The c.3857G>A variant leads to an arginine-to-histidine substitution at position 1286 of C5orf42 protein (p.R1286H), which is predicted to be probably damaging by PolyPhen-2 (with a score of 1.0) and deleterious by PROVEAN (with a score of -3.795). According to the ACMG variant classification guideline [9], the c.3857G>A variant could be classified as likely pathogenic (v) with 2 moderate (PM2, PM3) and 2 supporting (PP3, PP4) bodies of evidence.

## 4. Discussion

Reports on* in utero* diagnosis of JS are rare [15–24]. Prenatal ultrasound is the primary screening method for evaluation of posterior fossa abnormalities, and cranial MRI can be more helpful and provide more important information for the diagnosis of JS. Saleem et al. reported prenatal MRI diagnosis of JS in two unrelated fetuses as early as 17-18 weeks of gestation through detection of MTS [22]. To maximize the accuracy of prenatal diagnosis, Doherty et al. proposed a protocol for monitoring pregnancies at risk for JS, using serial ultrasounds in combination with MRI at 20-22 weeks of gestation [17]. In this study, the fetus was diagnosed with JS by ultrasound and MRI. Prenatal ultrasonographies performed at 23^+4^ and 29^+3^ weeks of gestation both revealed vermian hypoplasia of the fetus, which was consistent with the MTS identified by MRI at 29^+3^ weeks of gestation.

In 2012, Srour et al. reported that* C5orf42* is a causative gene of JS in the French Canadian population [25]. Human* C5orf42* gene contains 52 exons encoding a protein of 3197 amino acids, and the C5orf42 protein was highly conserved among other vertebrates and predicted to be a transmembrane protein with two transmembrane domains and two coiled coil domains [13].* C5orf42* gene is expressed in a variety of tissues including brain, but little is known about its function [25]. Lopez et al. identified a total of 14 novel* C5orf42* variants in 9/11 families with oral-facial-digital syndrome type VI (OFD VI) in 2013 and concluded that* C5orf42* is the major gene responsible for OFD VI[26]. In 2015, Romani et al. identified* C5orf42 *variants in 28 of 313 JS probands (8.9%)[13]. Bachmann-Gagescu et al. sequenced 27 JS-related genes in 375 families with JS in 2015 and identified causative variants in 62% of families, in which* C5orf42* variants account for 6-9% of JS and were highly correlated with polydactyly (OR 2.7, CI 1.2-5.9; p=0.01) [27]. In 2016, 51 Northern European JB cases were genotyped for 22 known JS-correlated genes and 599 additional ciliary genes by Kroes et al., and the results revealed that* C5orf42* variants were the most prevalent (12%)[28]. Vilboux et al. identified the causative genes in 94% of the families (81/86) with JS using a targeted panel of 27 JS-associated genes followed by whole-exome sequencing (WES) in 2017, and* C5orf42* variants were the most common variants in the JS patients with polydactyly [29].

In this study, two compound heterozygous variants, c.3599C>T and c.3857G>A, were identified in* C5orf42 *gene of the fetus diagnosed with JS, which are inherited from the mother and father respectively. The missense variant (c.3599C>T) has been reported in multiple patients with JS [12–14] and classified as pathogenic in the ClinVar database (https://www.ncbi.nlm.nih.gov/clinvar/variation/217591/). Although the c.3857G>A variant is present in dbSNP, ExAC, gnomAD, the 1000 Genomes Project database, and the database of Chinese genomes in diseaseDX, the allele frequency was extremely low and never in the homozygous state. The c.3857G>A variant resulted in the substitution of arginine for histidine at position 1286, which is predicted as damaging. In addition, the arginine residue (R1286 in the human protein) resides in a region highly conserved among species (Figure 2(c)), and the neighboring missense variant p.D1287H was found in compound heterozygous state in two sib fetuses with OFD VI by Lopez et al. [26] and classified as pathogenic in the ClinVar database (https://www.ncbi.nlm.nih.gov/clinvar/variation/157516/). Furthermore, the fetus with* C5orf42* variants had polydactyly, which is consistent with the results of previous reports [28, 29].

Since the prognosis of JS could be poor, prenatal diagnosis is necessary for the families with history of JS. The proband's parents have sought genetic counseling in our center, and prenatal diagnosis of a subsequent pregnancy was performed. The results of prenatal ultrasound and MRI examinations were normal (data not shown), and prenatal genetic analysis using amniotic fluid revealed that the fetus did not carry the variants identified in the proband (Figure 2(b)). A healthy boy was born without complications.

In conclusion, we report the prenatal diagnosis of a fetus with JS by ultrasound and MRI examinations and identification of a known missense variant and a novel missense variant in the* C5orf42* gene of the fetus. Our findings emphasize the role of ultrasound and MRI in the prenatal diagnosis of JS and broaden the variation spectrum of* C5orf42* in JS.

## Data Availability

The data used to support the findings of this study are included within the article.
